# Supplementary material for: Mathematical relationships between spinal motoneuron properties
Source: eLife. 2022 Jul 18;11:e76489. doi: 10.7554/eLife.76489 (PMC9612914; doi:10.7554/eLife.76489)
Supplement: Transparent reporting form [file elife-76489-transrepform1.docx]

***eLife’s* transparent reporting form**

We encourage authors to provide detailed information *within their submission* to facilitate the interpretation and replication of experiments. Authors can upload supporting documentation to indicate the use of appropriate reporting guidelines for health-related research (see [EQUATOR Network](http://www.equator-network.org/%20)), life science research (see the [BioSharing Information Resource](https://biosharing.org/)), or the [ARRIVE guidelines](http://www.plosbiology.org/article/info:doi/10.1371/journal.pbio.1000412) for reporting work involving animal research. Where applicable, authors should refer to any relevant reporting standards documents in this form.

If you have any questions, please consult our Journal Policies and/or contact us: [editorial@elifesciences.org](mailto:editorial@elifesciences.org).

**Sample-size estimation**

- You should state whether an appropriate sample size was computed when the study was being designed
- You should state the statistical method of sample size computation and any required assumptions
- If no explicit power analysis was used, you should describe how you decided what sample (replicate) size (number) to use

Please outline where this information can be found within the submission (e.g., sections or figure legends), or explain why this information doesn’t apply to your submission:

In this study we processed data from published literature, so the sample size was determined by the data we could retrieve through manual digitisation of figures in manuscripts and obtain through communication with the authors of the publications. The study search and selection criteria are described in the first subsection of Methods section of the study (“Studies reporting processable in vivo adult mammal data”). In total, we obtained around 4000 data points of pairs of motoneuron and/or muscle unit electrophysiological and/or morphometric properties from 40 experimental studies.

**Replicates**

- You should report how often each experiment was performed
- You should include a definition of biological versus technical replication
- The data obtained should be provided and sufficient information should be provided to indicate the number of independent biological and/or technical replicates
- If you encountered any outliers, you should describe how these were handled
- Criteria for exclusion/inclusion of data should be clearly stated
- High-throughput sequence data should be uploaded before submission, with a private link for reviewers provided (these are available from both GEO and ArrayExpress)

Please outline where this information can be found within the submission (e.g., sections or figure legends), or explain why this information doesn’t apply to your submission:

No experiment was conducted in the scope of this research, which used instead data published in previous literature. Details about the experiments that generated the datasets are available in the original publications.

We removed two points as outliers from the data digitised from Kanda & Hashizume (1992), as they were outside two standard deviations of the reported innervation ratio distribution. This is described in the section “Relationships between MN and mU properties” of the Results section.

**Statistical reporting**

- Statistical analysis methods should be described and justified
- Raw data should be presented in figures whenever informative to do so (typically when N per group is less than 10)
- For each experiment, you should identify the statistical tests used, exact values of N, definitions of center, methods of multiple test correction, and dispersion and precision measures (e.g., mean, median, SD, SEM, confidence intervals; and, for the major substantive results, a measure of effect size (e.g., Pearson's r, Cohen's d)
- Report exact p-values wherever possible alongside the summary statistics and 95% confidence intervals. These should be reported for all key questions and not only when the p-value is less than 0.05.

During this research we did not conduct any experiment, but used existing data published in the literature.

As described in the Methods section (“Relationships between MN properties” subsection), the raw data was normalized as percentage of the maximum recorded value for the property under evaluation, to improve inter-study consistency. All raw and normalised data are provided as source files.

Following the methodology described in the Methods (“Normalized space”, “Size-dependent normalized relationships”, “Normalized mathematical relationships between electrophysiological properties”), the normalised datasets were fitted using power functions. The coefficients of determination and p-values for these relationships are reported in Table 3, 7 and 8.

The relationships were finally validated using a k-fold cross-validation approach, reporting the normalized maximum error, normalized root-mean-square error and coefficient of determination (Figure 6).

Please outline where this information can be found within the submission (e.g., sections or figure legends), or explain why this information doesn’t apply to your submission:

(For large datasets, or papers with a very large number of statistical tests, you may upload a single table file with tests, Ns, etc., with reference to sections in the manuscript.)

**Group allocation**

- Indicate how samples were allocated into experimental groups (in the case of clinical studies, please specify allocation to treatment method); if randomization was used, please also state if restricted randomization was applied
- Indicate if masking was used during group allocation, data collection and/or data analysis

Please outline where this information can be found within the submission (e.g., sections or figure legends), or explain why this information doesn’t apply to your submission:

Only data obtained from studies measuring motoneuron and muscle unit properties in adult mammals using a similar experimental protocol were selected (see Methods, first section). The data were grouped by species (cat, rat and mice), and for each species, further subdivided by pairs of electrophysiological or morphological properties. The pairs are represented in Figure 2, the cat data in Figure 3 and Figure 4, the rat, and mice data in Figure 7. It is worth mentioning that in the validation phase, each sub-dataset was randomly shuffled and further split into a training set and a test set.

**Additional data files (“source data”)**

- We encourage you to upload relevant additional data files, such as numerical data that are represented as a graph in a figure, or as a summary table
- Where provided, these should be in the most useful format, and they can be uploaded as “Source data” files linked to a main figure or table
- Include model definition files including the full list of parameters used
- Include code used for data analysis (e.g., R, MatLab)
- Avoid stating that data files are “available upon request”

Please indicate the figures or tables for which source data files have been provided:

The manually digitised data from published figures, for which public datasets were not available, are shared as source data for each figure where they are presented, including both the raw and the normalised data.

The normalised relationships obtained from the normalised datasets are also shared as supplementary material.
